# Multiple Pickering emulsions stabilized by the same particles with different extent of hydrophobization *in situ*

**DOI:** 10.3389/fchem.2022.950932

**Published:** 2022-08-19

**Authors:** Yue Zhu, Tingting Chen, Zhenggang Cui

**Affiliations:** ^1^ School of Chemistry and Chemical Engineering, Nantong University, Nantong, China; ^2^ The Key Laboratory of Synthetic and Biological Colloids, Ministry of Education, School of Chemical and Material Engineering, Jiangnan University, Wuxi, China

**Keywords:** multiple emulsions, CaCO_3_ nanoparticles, hydrophobization *in situ*, anionic surfactant, stabilization

## Abstract

Multiple emulsions are widely used in pharmaceuticals, foods, and cosmetics. However, those stabilized by surfactants with different HLB values are generally unstable due to the diffusion of the surfactants between inner and outer interfaces. Here, we report that multiple W/O/W emulsions can be prepared by using the same particles in combination with a surfactant of different concentrations. The less surface-active raw CaCO_3_ nanoparticles can be hydrophobized to surface-active *in situ* by adsorption of the anionic surfactant SDS, and the wettability of the particles can be controlled to be suitable for stabilizing both O/W and W/O Pickering emulsions by adjusting the surfactant concentration. With toluene as oil phase, the CaCO_3_ particles at 1.0 wt% tend to stabilize a W/O emulsion in the presence of 3 mm SDS in an aqueous solution, which can then be further dispersed in an aqueous phase with 1.0 wt% CaCO_3_ and SDS below 1 mm to form a W/O/W multiple emulsion. The effects of the ratio of W/O emulsion to the outer water phase and the preparation methods on stabilization of multiple emulsions were examined. With a ratio smaller than 3:1 and by gentle magnetic stirring, the multiple emulsions obtained can stay stable for at least a month without coalescence. This simple method not only ensures stabilization of multiple emulsions but also avoids complicated synthesis of colloid particles with different wettability.

## 1 Introduction

Emulsions are heterogeneous systems of two immiscible liquids, with one liquid dispersed in the other as droplets (Becher, 1983). The immiscible liquids involved are mostly oil and water; therefore, emulsions are normally either oil-in-water (O/W) or water-in-oil (W/O) types. For stabilization of emulsions, it is necessary to add emulsifiers including surfactants, amphiphilic polymers, and colloid particles, which can reduce the interfacial tension and endow droplets with charges or steric films through interfacial adsorption. While emulsions stabilized by surfactants are relatively unstable, those stabilized by amphiphilic colloid particles are ultrastable due to the high adsorption free energy of the particles at the fluid interface, which makes the adsorption almost irreversible ( Binks, 2002; Aveyard et al., 2003; Kruglyakov and Nushtayeva, 2004; Binks et al., 2006).

Multiple emulsions are complex systems with both O/W and W/O microstructures in one system, in which droplets of emulsions in the internal phase are dispersed in the external phase (Florence and Whitehill, 1982; Bonnet et al., 2010; Li et al., 2012; Cizauskaite et al., 2017; Panagopoulou et al., 2017). There are mainly two types of multiple emulsions, W/O/W and O/W/O types. In W/O/W multiple emulsions, W/O emulsion is additionally dispersed in a water phase, whereas in O/W/O emulsions, O/W emulsion is additionally dispersed in an oil phase. Multiple emulsions were first found by Seifritz in 1925 (Seifritz, 1925) and first used in drug vehicles in 1968 (Engel et al., 1968). In recent years, multiple emulsions have aroused great interest because they have wide applications in pharmaceuticals (Moussaoui et al., 2016), chemicals (Liu et al., 2017), foods (Lutz et al., 2009; Jiménezcolmenero, 2013), cosmetics (Hoppel et al., 2015; Wang et al., 2017), materials (Akbaba et al., 2017), and many other fields (Cournarie et al., 2004; Zhao et al., 2022). The preparation of multiple emulsions includes one-step and two-step approaches (Morais et al., 2009; Fernández-Martín et al., 2017), with the two-step method being more common (Fernández-Martín et al., 2017). The stability of multiple emulsions against coalescence is determined by several factors including internal/external phase volume ratio, hydrophobic/hydrophilic emulsifiers, and the species of oil phase (Taisne et al., 1996; Keshmiri et al., 2016; Zhao et al., 2022), in which emulsifiers play normally a critical role. Traditional multiple emulsions are obtained by using two surfactants of different HLB, one is hydrophilic and the other one is hydrophobic. For example, Cole and Whateley, (1997) demonstrated the effects of Pluronic F127 and polyacrylic acid (PAA) on the stability of multiple W/O/W emulsions (Cole and Whateley, 1997).

As mentioned above, emulsions stabilized by surfactants are thermodynamically unstable, and the multiple emulsions generated with surfactants as emulsifiers are similarly thermodynamically unstable due to the diffusion of two surfactants between the inner and outer interfaces, which aggravatingly accelerates droplet coalescence. For improving the stability of the multiple emulsions, many researchers have used additional substances such as polymers, microparticles, and nanoparticles (Riess and Labbe, 2004; Hong et al., 2012; Clegg et al., 2015; Maciel et al., 2015; Mozafari, 2015; Zamani et al., 2018; Ghasemi et al., 2020a; Ghasemi et al., 2020b; Saffarionpour and Diosady, 2021; Zhao et al., 2022). Particles as emulsifiers were considered better than surfactants because of their firmer adsorption at the interface, which constitutes a solid obstacle against coalescence. However, most of these particles are functional polymers, and their synthesis is relatively complex.

CaCO_3_ nanoparticles are the commercial nanoparticles with probably the highest output and cheapest price in the world. Moreover, they are edible and friendly to the environment, which leads to their wide use in the fields of plastics, paper, medicine, food products, and so on. In previous studies, it has been found that although natural particles have no strong surface activity, they can be *in situ* hydrophobized by absorption of surfactants with opposite charges to become surface active (Cui et al., 2008; Cui et al., 2012). Different from *ex situ* hydrophobization, where chemical modifications are usually involved, *in situ* hydrophobization is a physical process in solution, where the wettability of the particles is modified by adsorption of the surfactant *via* electrostatic interaction, and the wettability can be adjusted by means of the concentration and structure of the surfactant (Cui et al., 2008). Herein, we report that Pickering multiple emulsions can be prepared using the same materials (CaCO_3_ nanoparticle and surfactant) as emulsifiers for the internal and external interfaces and demonstrate the behaviors of the multiple emulsions formed. The use of *in situ* surface-modified CaCO_3_ nanoparticles as an emulsifier is very convenient, which avoids the complicated synthesis of the functional particles.

## 2 Experimental

### 2.1 Materials

Calcium carbonate (CaCO_3_) nanoparticles with a purity of 98% were purchased from Keli New Materials Co. Ltd, Henan, China. Sodium dodecyl sulfate (SDS, 99%) was purchased from Sigma. All other chemicals including toluene (99.5%), HCl (≥99.5%), and NaOH (≥99.5%) were purchased from Sinopharm Chemical Reagent Co. Ultrapure water (resistance = 18.2 MΩ, pH = 6.8 at 25°C) was provided by Huawei Co. Ltd., Wuxi, China.

### 2.2 Methods

#### 2.2.1 Aqueous dispersion of CaCO_3_ particles

Powdered CaCO_3_ particles were weighted into a screw-cap glass vial [2.5(d) cm × 6.5 (h) cm], and then pure water or SDS solutions were added. The particles were then dispersed by using an ultrasound probe (FS-250N, Shanghai ShengXi Co.) operating at 50 W for 2 min to obtain dispersion 1.

#### 2.2.2 Preparation of W/O Pickering emulsions and W/O/W multiple emulsions

1) W/O Pickering emulsions.

7 ml dispersion 1 was transferred into a glass vial [2.5 (d) cm ×6.5 (h) cm], followed by the addition of 7 ml toluene, with oil/water volume ratio = 1:1. The mixture was then homogenized by using an ultraturrax homogenizer (IKA T18 basic) with S18N-10G head working at 7,000 rpm for 2 min.

2) W/O/W Pickering multiple emulsions.

Another dispersion (dispersion 2) of CaCO_3_ nanoparticles in SDS solutions with lower SDS concentration was prepared using a similar procedure as described above, which was transferred to a glass vial [2.5 (d) cm ×6.5 (h) cm], and then the W/O/W Pickering multiple emulsions were prepared by using two different methods as described below.

Method 1: The dispersion 2 was stirred using a magnetic stirrer (Guohua 76, CHangzhou Guohua Co.) operating at 1,400 rpm, and the W/O Pickering emulsions previously prepared were added drop by drop.

Method 2: Both dispersion 2 and the W/O Pickering emulsion were added to the glass vial at a certain volume ratio, and the mixture was then homogenized using the IKA T18 basic ultraturrax homogenizer at 3,500 rpm for 2 min.

#### 2.2.3 Characterization of the emulsions prepared

The type of the simple emulsion prepared was verified by the drop test (Cui et al., 2010a), and the photos of the simple/multiple Pickering emulsions were taken using a digital camera at different times for observing the stability of the emulsions. For observing the microstructure of the emulsions, emulsion samples were dropped on a glass slide followed by diluting with the continuous phase, and the micrographs of the simple/multiple Pickering emulsions were taken by using a VHX-1000 microscope system (Keyence Co.).

#### 2.2.4 Zeta potential and particle size

1 wt% CaCO_3_ nanoparticles were dispersed in pure water at different pH adjusted by HCl or NaOH at 25°C. The dispersions were then stood for about 24 h at the same temperature to reach equilibrium. The zeta potentials were then measured by using Zetasizer 2000 (Malvern) at 25°C. For measuring the size of CaCO_3_ nanoparticles, 1 wt% CaCO_3_ nanoparticles were dispersed in pure water and 1 mm SDS solution using the using ultrasound probe, and the size of the particles was measured using Zetasizer Nano-ZS90 (Malvern) at 25°C.

#### 2.2.5 Adsorption isotherm of SDS on CaCO_3_ nanoparticles

The dispersions with 0.4 g CaCO_3_ nanoparticles and 20 ml aqueous solution of SDS at different concentrations were prepared as described above and left in a thermostatic chamber overnight at 25°C. Then, the suspended particles were removed from the dispersion by centrifugation (5,000 rpm, 15 min). To determine the equilibrium concentration of SDS in the supernatant, a two-phase titration method (Cui et al., 2010b) was used, and the adsorbed amount (Γ) of SDS on the CaCO_3_ nanoparticles was calculated by:

$$\Gamma =\frac{V\left(C_{0}-C\right)}{w}\left(mmol/g\right),$$ (1)

where C_0_ and C are the initial and equilibrium concentrations (mol/L) of SDS in the solution, respectively; V is the volume (ml) of the SDS solution; and w is the weight (g) of the CaCO_3_ particles. At the equilibrium concentration of SDS below 0.5 mm, the equilibrium concentration of SDS was determined by a spectrophotometric method (Cui et al., 2008).

#### 2.2.6 Contact angles of SDS solution on a CaCO_3_ substrate

A piece of CaCO_3_ stone of 99 wt% was polished to be a smooth plane. The contact angles of drops of SDS solutions at different concentrations on the particle surface in the air were measured by Drop Meter-A-100, an optical drop shape analyzer from Ningbo HaishuMaishi Scientific Test Co. After one measurement (several drops) was finished, the surface of the CaCO_3_ stone was polished and washed for the next measurements.

## 3 Results and discussion

### 3.1 Characterization of CaCO_3_ nanoparticles

**FIGURE 1:**
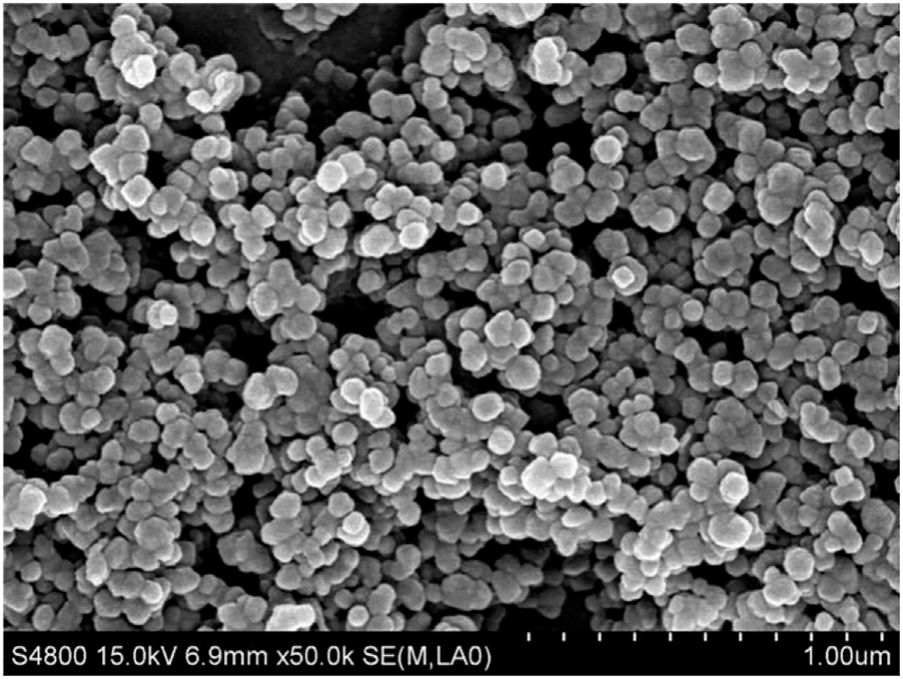
SEM image of CaCO_3_ nanoparticles.

**FIGURE 2:**
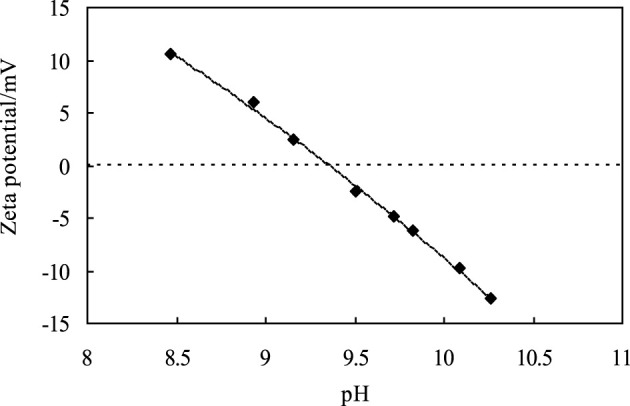
Zeta potential of 1.0 wt% CaCO_3_ nanoparticles in water of different pH.

The CaCO_3_ nanoparticles used are quasi-spherical that have a primary diameter 80–120 nm, according to the SEM image shown in Figure 1. The surface area is 16.21 m^2^/measured by BET. Assuming the particle is spherical, the calculated diameter is 71.16 nm, which is in good agreement with the SEM image. Figure 2 shows the zeta potential of the particles at different pH values, which decreases with increasing pH, and the bare CaCO_3_ nanoparticles have an isoelectric point of 9.35. At pH < 9.35, the CaCO_3_ particles are positively charged, and on the contrary, the particles are negatively charged. Therefore, the CaCO_3_ particles are positively charged when dispersed in pure water with the pH of dispersion being 8.93 (<9.35).

**FIGURE 3:**
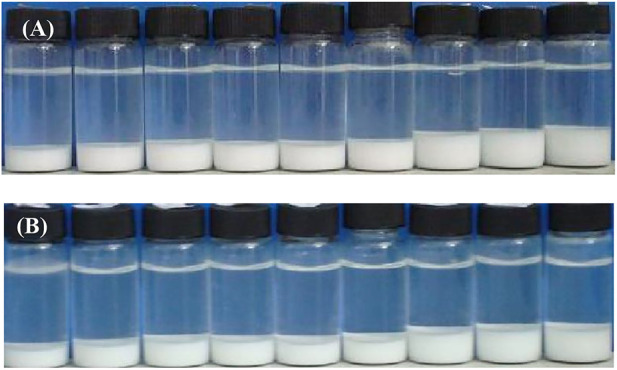
Appearance of 1.0 wt% CaCO_3_ nanoparticles dispersed in aqueous solutions with HCl or NaOH at different concentration (from left to right) (HCl)1 × 10^−2^,1 × 10^−3^,1 × 10^−4^, and 1 × 10^−5^ mol/L, water (NaOH)1 × 10^−5^,1 × 10^−4^,1 × 10^−3^, and 1 × 10^−2^ mol/L, taken 1 h **(A)** and 1 week **(B)** after preparation.

The appearance of 1 wt% CaCO_3_ nanoparticles dispersed in pure water at different pH is illustrated in Figure 3. It is seen that the particles are almost all sediment after 1 h, and the sedimentation layer of the particles increases as pH increases, which can be explained by the DLVO theory. Sedimentation or dispersion depends on the attraction and electrostatic repulsion between the particles. When the electrostatic repulsion is stronger, the distance between particles increases. Figure 3 suggests that at high pH, the increased zeta potential results in stronger repulsion and, therefore, a thicker sedimentation layer.

### 3.2 Pickering emulsions co-stabilized by CaCO_3_ nanoparticles and SDS

**FIGURE 4:**
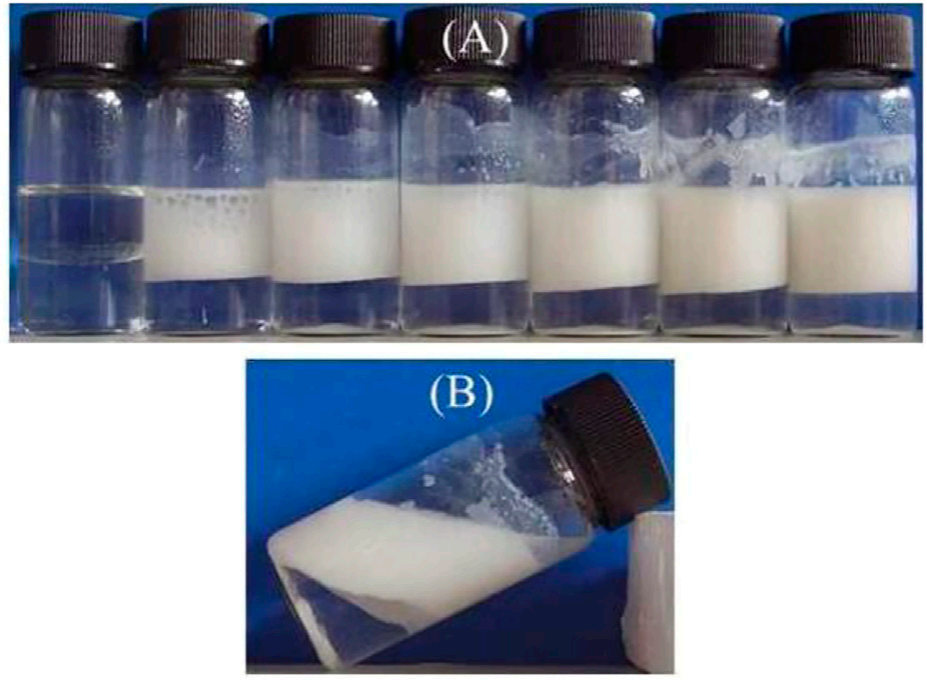
Appearance of toluene-in-water emulsions stabilized solely by CaCO_3_nanoparticles, taken 1 week after preparation. Particle concentration **(A)** left to right 0, 0.1, 0.3, 0.5, 1.0, 1.5, and 2.0 wt% **(B)** 1.0 wt%.

The emulsions (toluene as oil phase) formed by CaCO_3_ nanoparticles alone are not very stable. It seems to be stable from the appearance shown in Figure 4A, but coalesced oil appeared on the top of the vessels, which can be seen clearly after the emulsions were kept for 1 week, as shown in Figure 4B. This indicates that the bare CaCO_3_ nanoparticles are hydrophilic and weakly surface-active.

**FIGURE 5:**
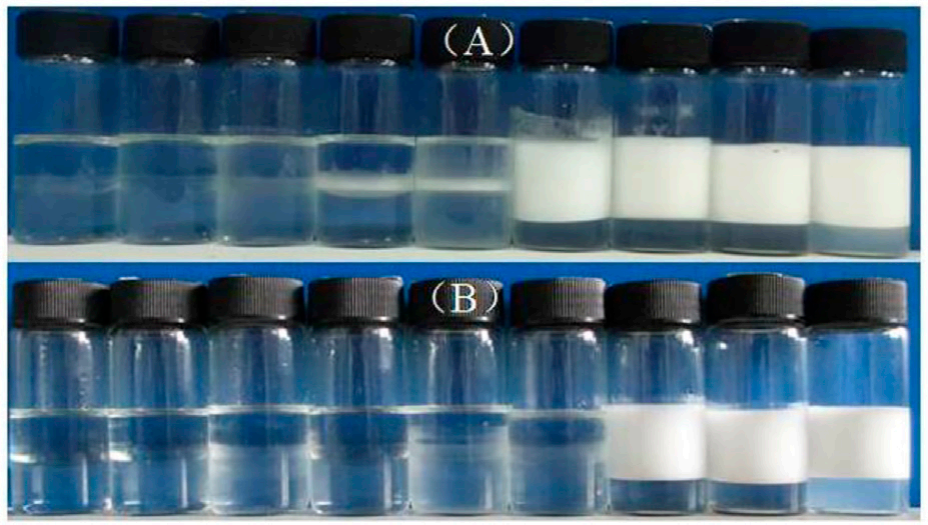
Photographs of toluene-in-water emulsions stabilized by SDS alone at different concentration, taken **(A)** 24 h and **(B)** 1 week after preparation. Concentration of SDS (from left to right):3 × 10^−5^, 6 × 10^−5^, 1 × 10^−4^, 3 × 10^−4^, 6 × 10^−4^, 1 × 10^−3^, 3 × 10^−3^, 6 × 10^−3^, and 1 × 10^−2^ mol/L.

**FIGURE 6:**
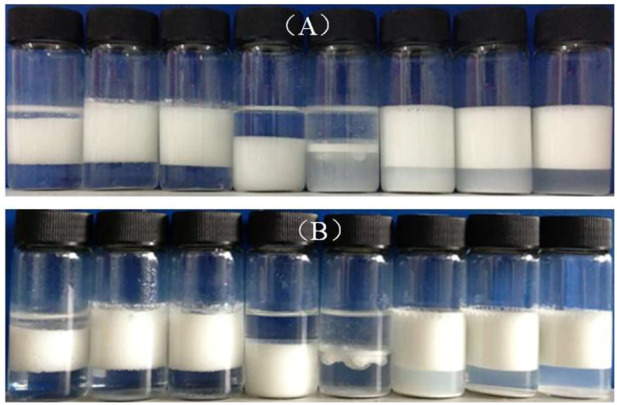
Appearance of toluene–water emulsions stabilized by 1.0 wt% CaCO_3_ nanoparticles together with SDS at different concentration (from left to right): 3 × 10^−4^, 6 × 10^−4^, 1 × 10^−3^ (O/W (1)), 3 × 10^−3^ (W/O), 6 × 10^−3^ (unstable), 1 × 10^−2^, 3 × 10^−2^, and 6 × 10^−2^ [O/W (2)] mol/L, taken **(A)** 24 h and **(B)** 1 week after preparation.

**FIGURE 7:**
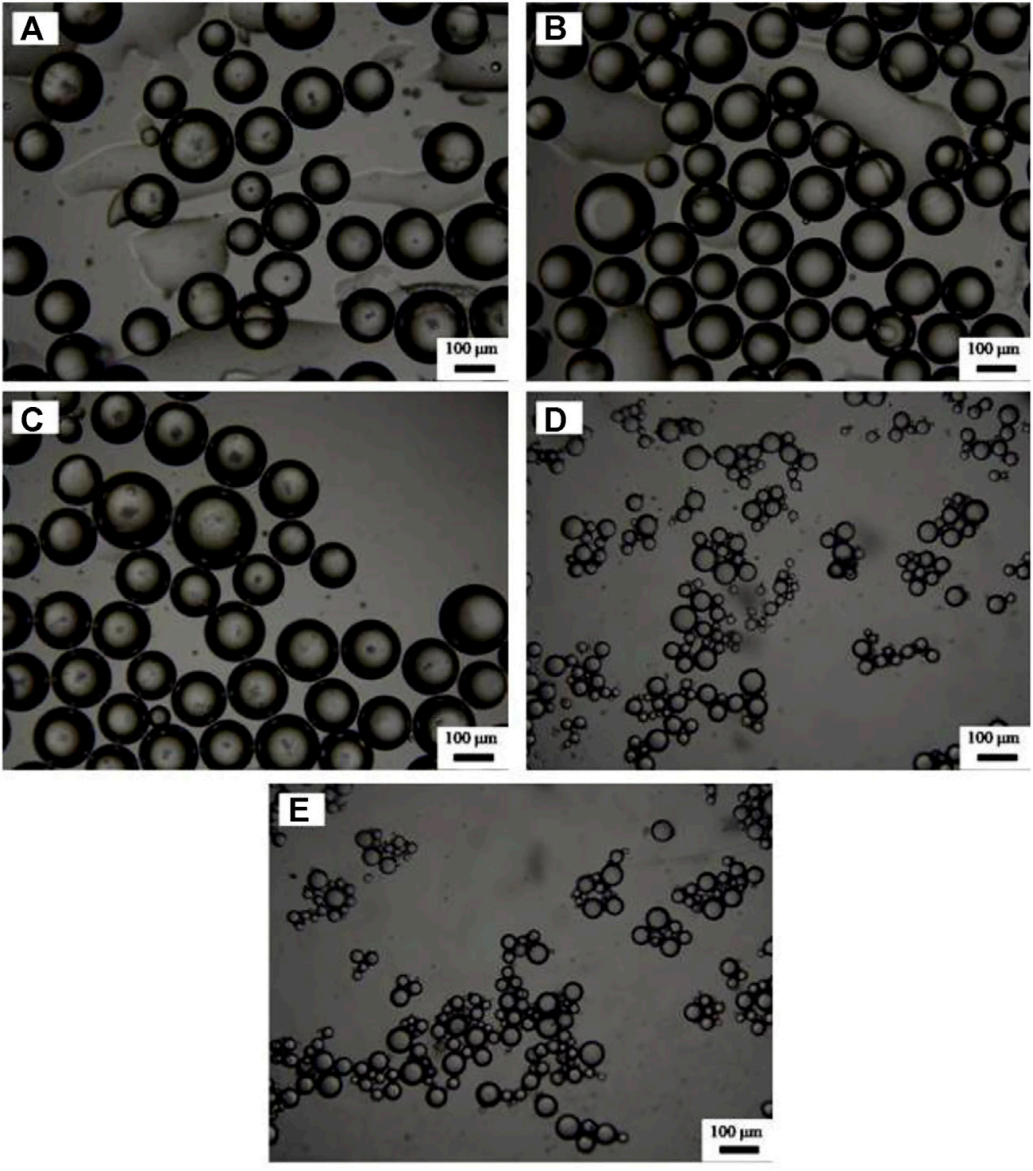
Optical micrographs of emulsions stabilized by **(A–D)** 1.0 wt% CaCO_3_ nanoparticles and SDS of various concentrations and by **(E)** SDS solely, taken 24 h after preparation. The concentration of SDS (from A to E) is 0.6, 1, 3, 30, and 10 mm.

Figure 5 shows that using SDS alone as an emulsifier under 3 mm, no stable emulsion can be obtained. However, once CaCO_3_ nanoparticles and SDS solution coexist, toluene-in-water emulsions were formed, which kept still stable after a week. Meanwhile, double-phase inversion (Cui et al., 2008) of O/W (1)→W/O→O/W (2) was observed by increasing SDS concentration, as shown in Figure 6. When the concentration of SDS is lower than 1 mm, all the emulsions are O/W type Pickering emulsions, recorded as O/W (1), and when the concentration of SDS increases to 3 mm, the oil phase reverts to be continuous phase, leading to formation of W/O Pickering emulsions. Finally, when the concentration of SDS is beyond 10 mm, the second phase inversion of W/O→O/W (2) takes place, where the O/W (2) emulsion is stabilized by SDS. The micrographs in Figure 7 show that the average droplet sizes of O/W (1) and W/O emulsions have little difference but are much bigger than those stabilized by SDS alone and of O/W (2) emulsions, which suggests that both O/W (1) and W/O emulsions are stabilized by the *in situ* hydrophobized particles instead of SDS molecules, while O/W (2) emulsions are stabilized mainly by SDS, where the concentration is over its CMC.

### 3.3 Preparation and characterization of W/O/W multiple emulsions stabilized by CaCO_3_ nanoparticles in combination with SDS

**FIGURE 8:**
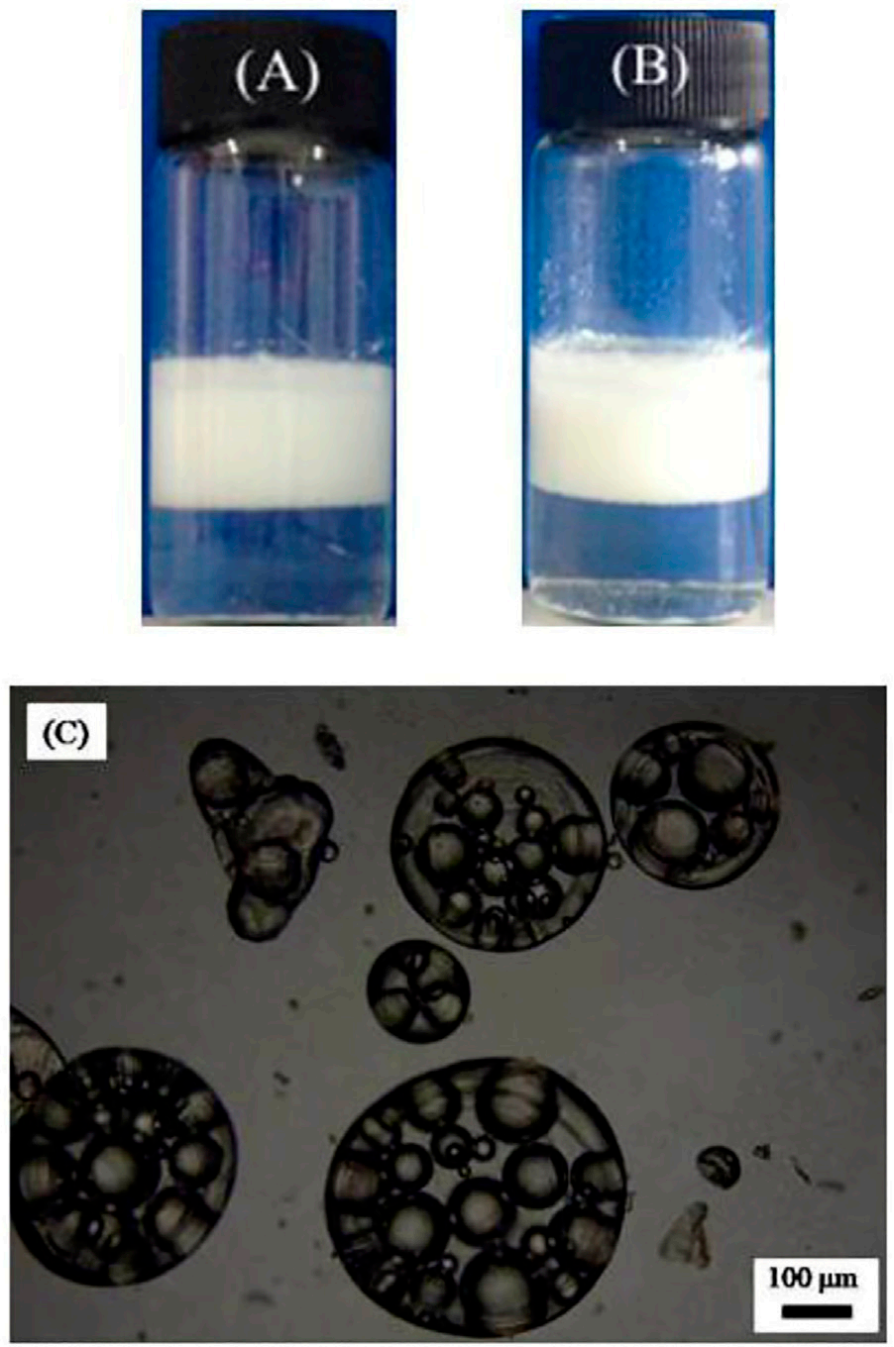
Appearance of the water-in-toluene-in-water multiple emulsion, taken **(A)** 1 day, **(B)** 1 week after preparation, and **(C)** optical micrograph recorded 24 h after preparation. The inner W/O emulsion is stabilized by 1.0 wt% CaCO_3_ nanoparticles and 3 × 10^−3^ mol/L SDS, and the outer O/W emulsion is stabilized by 1.0 wt% CaCO_3_ nanoparticles and 3 × 10^−4^ mol/L SDS. Volume ratio of inner W/O emulsion to the outer water phase = 2:1.

A W/O emulsion including 1.0 wt.% CaCO_3_ nanoparticles and 3 mm SDS was chosen as the internal phase, which was then added to an aqueous dispersion with 1.0 wt% CaCO_3_ nanoparticles and 0.1 mm SDS, at a volume ratio of 2:1. Subsequently, the two phases were mixed by a magnetic stirrer to obtain W/O/W multiple emulsions. The appearance of the multiple emulsions showed no change after 24 h and 1 week, as shown in Figures 8A,B. The micrographs in Figure 8C proved that multiple emulsion has been formed, where a big outer droplet contains several small inner droplets. Also, the diameter of the external droplets of around 400 μm was much bigger than those stabilized by the surfactants alone.

**FIGURE 9:**
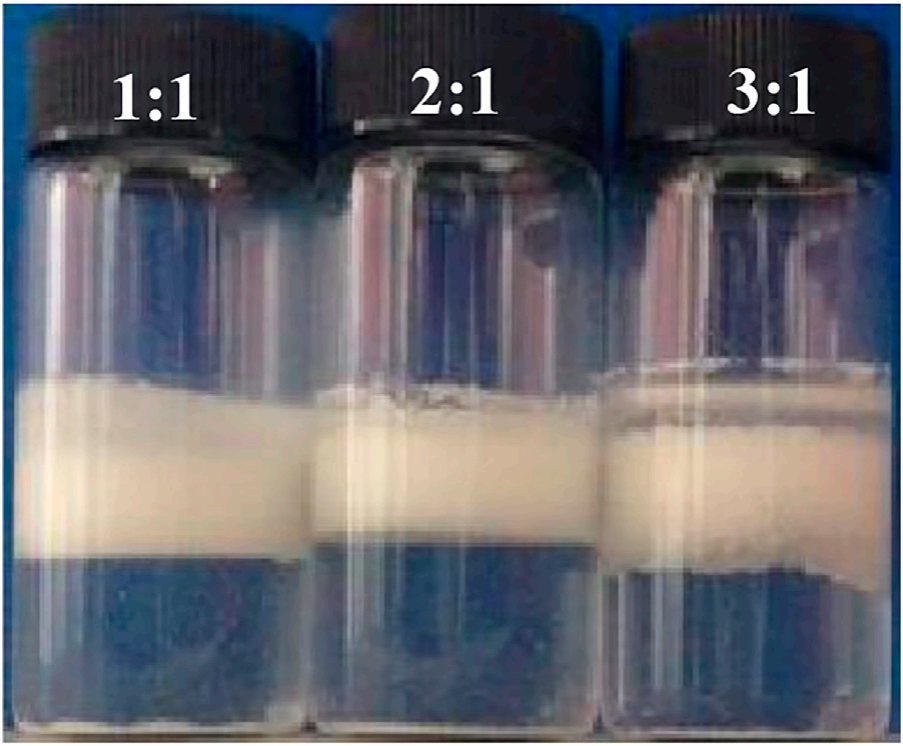
Appearance of the water-in-toluene-in-water multiple emulsions, taken 1 week after preparation. The inner W/O emulsion is stabilized by 1.0 wt% CaCO_3_ nanoparticles in combination with 3 × 10^−3 ^mol/L SDS, and the outer aqueous phase is stabilized by 1.0 wt% CaCO_3_ nanoparticles together with 3 × 10^−4 ^mol/L SDS. The volume ratio of inner water-in-toluene/outer aqueous phase is shown in the legend.

The influence of the volume ratio of the inner phase (W/O emulsion) to the outer phase (water phase) on the stability of the multiple emulsions was then studied. It is observed that when the volume ratio was 1:1 and 2:1, the multiple emulsions can be stable for 1 week, whereas with increasing the volume ratio to 3:1, the multiple emulsion became unstable with coalesced oil appeared, as shown in Figure 9.

**FIGURE 10:**
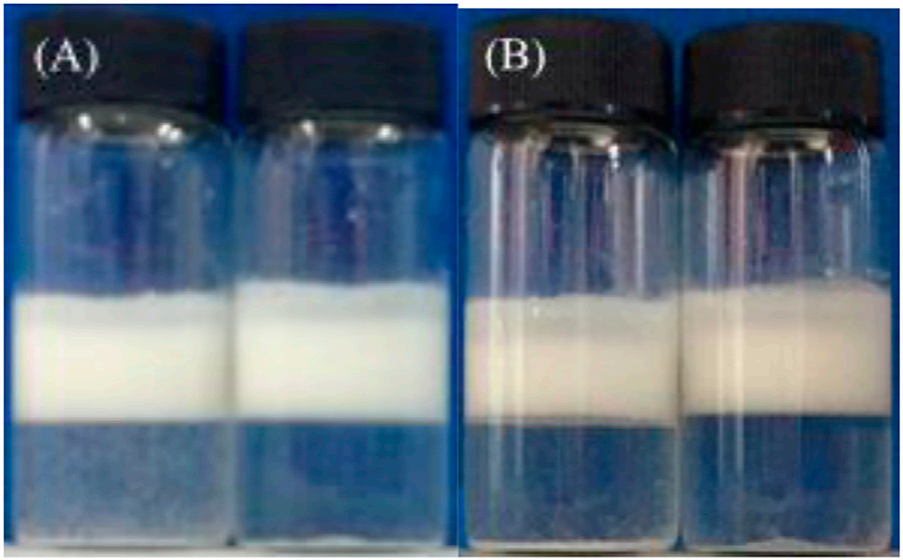
Appearance of the water-in-toluene-in-water multiple emulsions, taken **(A)** 1 day and **(B)**1 week after preparation by magnetic stirring (left) and homogenization (right). The inner W/O emulsion is stabilized by 1.0 wt% CaCO_3_ nanoparticles in combination with 3 × 10^−3 ^mol/L SDS, and the outer water phase contains 1.0 wt% CaCO_3_ nanoparticles and 3 × 10^−4 ^mol/L SDS. The volume ratio in inner water-in-toluene emulsion is 1:1, and the volume ratio of inner water-in-toluene emulsion to outer water phase is 1:1.

**FIGURE 11:**
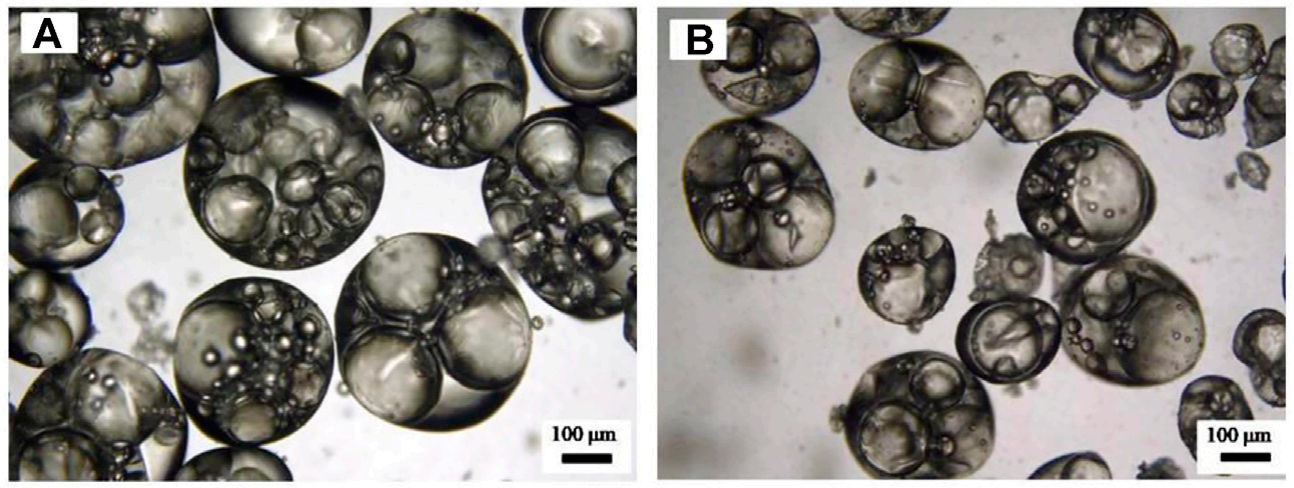
Micrograph of the water-in-toluene-in-water (W/O/W) multiple emulsions shown in Figure 10 prepared by **(A)** magnetic stirring and **(B)** homogenization, taken 24 h after preparation.

In addition, the influence of preparation methods (magnetic stirrer and homogenization) on multiple emulsions was also explored. It was noticed that although the appearance was almost not different (Figure 10), the oil droplets in the emulsion prepared by a magnetic stirrer were bigger, and each contains several water droplets, compared with that in emulsions prepared by homogenization, where oil droplets were relatively smaller, and each contains only 1–2 water droplets (Figure 11).

### 3.4 Surface activation mechanism of CaCO_3_ nanoparticles by *in situ* hydrophobization

**FIGURE 12:**
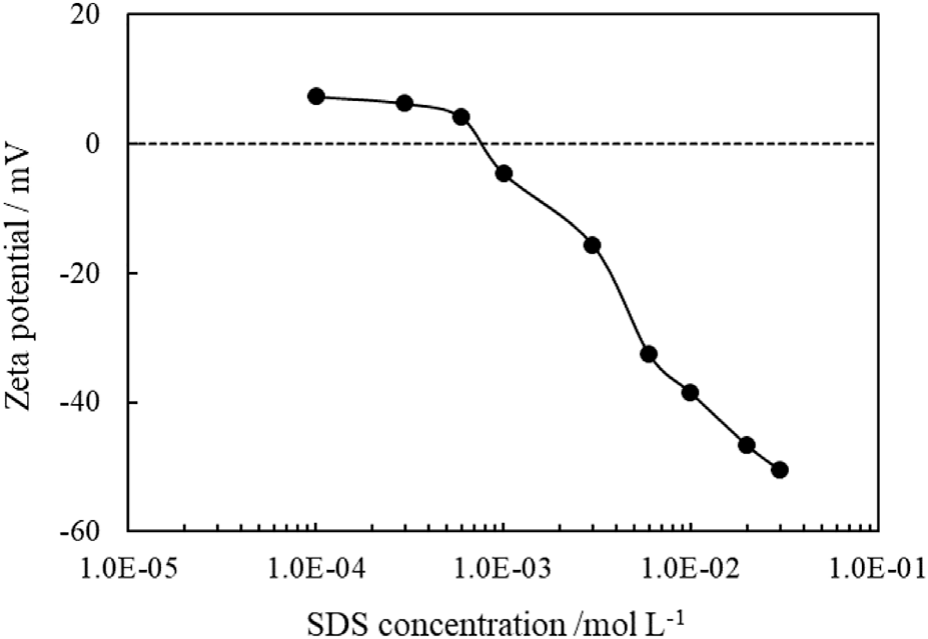
Zeta potential of 1.0 wt% CaCO_3_ nanoparticles dispersed in SDS solutions of different concentration at 25°C.

**FIGURE 13:**
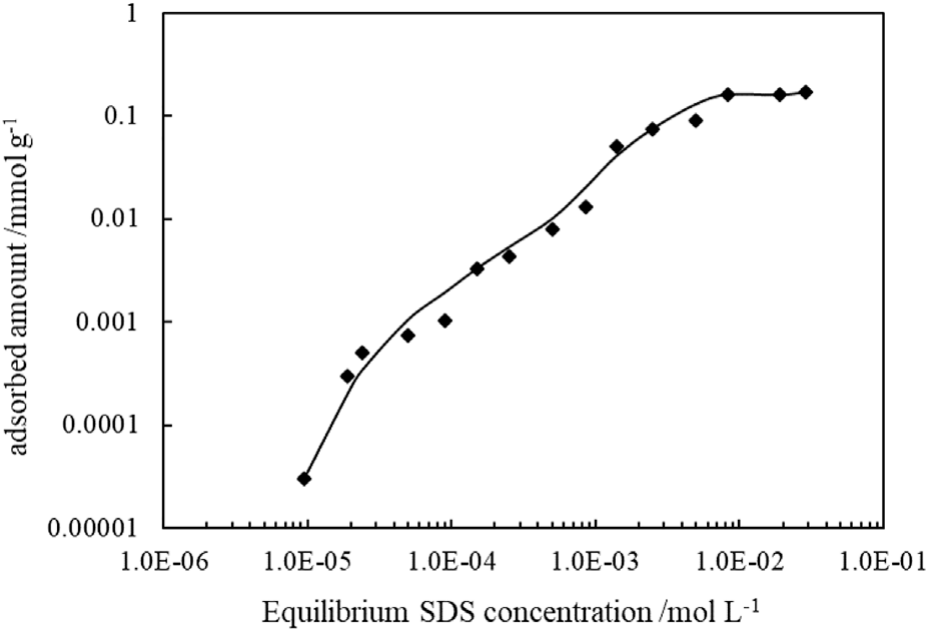
Adsorption isotherms of SDS at the CaCO_3_nanoparticle/water interface at 25°C.

It is known that ionic surfactant can adsorb on oppositely charged particle surfaces *via* electrostatic attraction, and by the formation of a monolayer, the hydrophilicity of particles surface is reduced. The adsorption of SDS on CaCO_3_ particles can be proved by the change of zeta potentials of the 1.0 wt% CaCO_3_ particles dispersed in SDS solutions of varied concentrations, as shown in Figure 12, and the adsorption isotherms are demonstrated in Figure 13. As the concentration of SDS increases, the charges on the particles are neutralized with the zeta potential changing from positive to negative, and the adsorption amounts increase, indicating adsorption of SDS molecules through electrostatic interaction. The adsorption of SDS on particle surfaces with hydrocarbon chain toward water modifies the wettability of CaCO_3_ nanoparticles. At lower SDS concentration (below 1 mm), the particles are weak hydrophobic, tending to stabilize O/W emulsion. However, at middle SDS concentration (3 mm), the particle surface turns more hydrophobic suitable to stabilize W/O Pickering emulsions.

**FIGURE 14:**
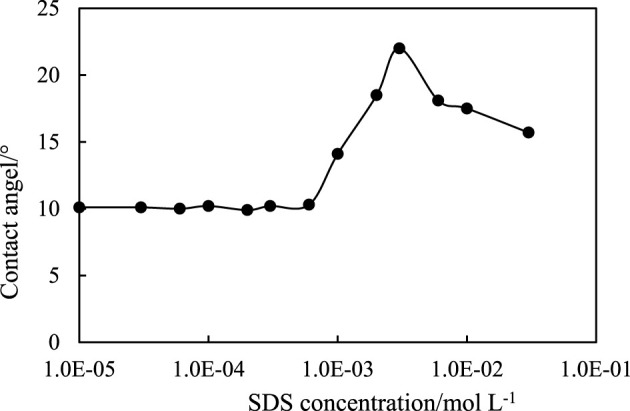
Contact angle of SDS aqueous solutions on CaCO_3_ plane in air at 25°C.

The configuration of the adsorbed SDS at the particle surface can be determined by the contact angle measurement, as shown in Figure 14. It is noticed that the contact angle of pure water on the CaCO_3_ surface is as low as 10°, whereas the contact angle of the SDS solution increases with increasing SDS concentration, reaching a maximum around 23° at an SDS concentration of 3 mm, and it is believed that the maximum contact angle of the SDS solution on CaCO_3_ surface in oil (toluene) should be much larger, or >90°, so that particles are hydrophobic enough to stabilize W/O emulsions. With further increasing SDS concentration, the contact angle decreases due to bilayer formation, which makes particle surface hydrophilic again since the second layer has an opposite configuration. It is then suggested that in the region of O/W (1) and W/O, the SDS molecules form a monolayer on particle surfaces with their head groups toward particle and hydrocarbon chains toward water, whereas in the region of O/W (2), SDS molecules form a bilayer on the CaCO_3_ particles, making the particles hydrophilic again with their head groups toward water.

**FIGURE 15:**
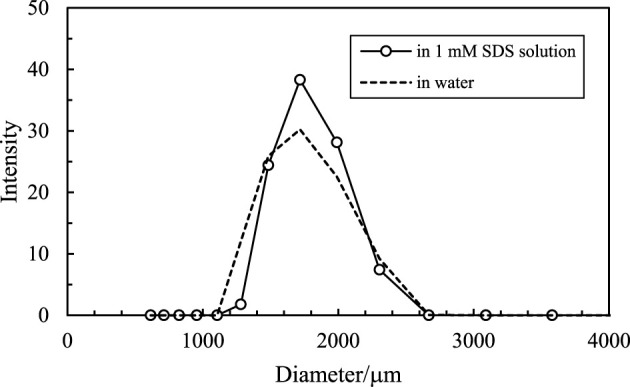
Size distribution of 1 wt% CaCO_3_ nanoparticles dispersed in water and 1 mm SDS solutions at 25°C.

It is noticed that the size of the oil droplets and the inner water droplets is relatively larger, as shown in Figure 11. This is because the CaCO_3_ nanoparticles aggregate to relatively larger aggregations in either water or SDS solution, as shown in Figure 15, although they have a small primary size (Figure 1). It is suggested that using nanoparticles with smaller sizes may be beneficial to obtain multiple Pickering emulsions with smaller droplet sizes.

## 4 Conclusion

A simple method is demonstrated to be effective for preparing multiple W/O/W emulsions by using the same particle (CaCO_3_ nanoparticles) as emulsifiers of both the internal and external interphases. The raw CaCO_3_ nanoparticles, which are less surface-active, can be made more surface-active by *in situ* hydrophobization *via* adsorption of an oppositely charged surfactant (SDS) and can then self-assemble at the fluid interface to stabilize simple Pickering emulsions and multiple Pickering emulsions. In addition, the wettability of the particles can be adjusted by controlling the SDS concentration, and phase inversion can then occur. At 3 mm SDS in an aqueous solution, the particles tend to stabilize W/O emulsion, whereas, at lower SDS concentrations (below 1 mm), the particles tend to stabilize O/W emulsion. Multiple emulsions can, therefore, be prepared using the same particles in combination with different concentrations of SDS. The multiple emulsions stabilized by CaCO_3_ nanoparticles can stay stable for at least a month avoiding coalescence, which makes them more stable compared with those stabilized by surfactants of different hydrophilicity. This protocol avoids complicated synthesis of the particles with different hydrophilicity and has potential applications in the fields of pharmaceuticals, foods, and cosmetics, where stable Pickering of multiple emulsions is expected.

## Data Availability

The original contributions presented in the study are included in the article/Supplementary Material; further inquiries can be directed to the corresponding authors.
